# Music-induced cognitive change and whole-brain network flexibility: a pilot study

**DOI:** 10.3389/fnins.2025.1567605

**Published:** 2025-06-05

**Authors:** E. Lydia Wu-Chung, Melia E. Bonomo, Anthony K. Brandt, Bryan T. Denny, Christof Karmonik, J. Todd Frazier, Karl Blench, Christopher P. Fagundes

**Affiliations:** ^1^Department of Psychology, University of Pittsburgh, Pittsburgh, PA, United States; ^2^Department of Physics and Astronomy, Rice University, Houston, TX, United States; ^3^Shepherd School of Music, Rice University, Houston, TX, United States; ^4^Department of Psychological Sciences, Rice University, Houston, TX, United States; ^5^Translational Imaging Center, Houston Methodist Research Institute, Houston, TX, United States; ^6^Center for Performing Arts Medicine, Houston Methodist Hospital, Houston, TX, United States; ^7^Department of Behavioral Science, The University of Texas MD Anderson Cancer Center, Houston, TX, United States; ^8^Department of Psychiatry & Behavioral Sciences, Baylor College of Medicine, Houston, TX, United States; ^9^Institute of Health Resilience and Innovation, Rice University, Houston, TX, United States

**Keywords:** creativity, fMRI, flexibility, network analysis, cognition, mild cognitive impairment, music intervention, older adulthood

## Abstract

**Introduction:**

Cognitive impairment that exceeds age-related cognitive decline is a risk factor for Alzheimer’s disease and related dementias. As the older adult population is notably increasing every year, significant efforts are being made to preserve cognitive function in older adulthood. Non-pharmaceutical approaches such as music interventions have noticeable benefits for cognition. Music engagement utilizes multiple brain regions dually involved in higher cognitive functions. Yet the neurobiology of music-induced cognitive change remains understudied. Complex human behavior and cognition likely depend on continuous communication across brain regions rather than localized activity in one region. Given that music creativity engages a wide range of mental processes, whole-brain network indices quantifying the brain’s tendency to create functional communities (modularity) and then dynamically reorganize these communities (flexibility) may be relevant for assessing music-related cognitive change. Using a semi-randomized clinical trial design (ClinicalTrials.gov; NCT04137913), we examined whether (1) music creativity altered whole-brain network indices (modularity, flexibility) and (2) whether music-related effects on cognition depended on whole-brain network indices.

**Methods:**

Fifty-two older adults (Mean age = 75 years; 54% female; 84% White) were randomized to a 6-week music creativity intervention (*n* = 25) or a no-treatment control condition (*n* = 27) and completed resting-state fMRI scans and the Montreal Cognitive Assessment at baseline and follow-up (post-intervention).

**Results:**

The music creativity intervention did not alter network flexibility or modularity over time. However, the relationship between group assignment and change in global cognitive function depended on baseline flexibility: music creativity improved global cognition more than the control condition, only among individuals who had higher than average network flexibility.

**Discussion:**

Findings suggest that having a dynamic brain network, which has previously been linked to better executive functioning performance, may be necessary for music-related benefits on cognition. This pilot study is innovative as it is among the first to identify possible neural mechanisms underlying why music creativity interventions confer a more significant cognitive benefit for some older adults than others.

## 1 Introduction

Cognitive impairment that exceeds normative age-related cognitive changes affects 15–20% of the population (Bai et al., 2022; Overton et al., 2019), with prevalence rates highest for those aged 65–85 years old (Ward et al., 2012). As the proportion of older adults is projected to double by 2050, society is increasingly invested in strategies to protect brain health and cognitive function.

Interventions involving the musical arts are promising, non-pharmaceutical approaches for people with cognitive impairment (Xu et al., 2017). In particular, music interventions that require active participation (e.g., music-making activities) as opposed to passive engagement (e.g., listening to music), produce noticeable benefits on global cognition in community and patient samples (Fusar-Poli et al., 2018; Gómez-Gallego et al., 2021; Wu-Chung et al., 2023). However, effect sizes remain small (Dorris et al., 2021), adequate control groups preclude assumptions of causality (Sutcliffe et al., 2020), and findings are oftentimes mixed (Fusar-Poli et al., 2018). Few studies investigate why and how music improves cognitive function. As a cognitively demanding activity, musical training is thought to mitigate age-related neurocognitive changes in two ways: by promoting brain plasticity (the brain’s ability to alter its structure and function in response to external and internal stimuli) (Fauvel et al., 2013) or by enhancing cognitive reserve (cognitive maintenance in the midst of aging, brain damage, or disease) (Stern, 2009). However, few studies include neuroimaging measures designed to assess change in functional brain health. Identifying neural mechanisms of change may help researchers and clinicians develop efficacious music interventions and discern who may benefit most from these interventions.

Music engagement is a multi-sensory experience involving several brain regions dually involved in cognitive processes (Särkämö et al., 2013). For example, music perception of rhythm, chords, and harmony engages the medial and inferior prefrontal cortex, anterior and posterior parts of the temporal gyrus, inferior parietal, and premotor cortex (Särkämö et al., 2013). Music improvisation, or creating novel notes and sequences in a short time span, involves the premotor cortex, the dorsolateral prefrontal cortex, and medial temporal regions (Bashwiner, 2018). These brain regions participate in a broad range of functions including executive function (e.g., medial prefrontal cortex, dorsolateral prefrontal cortex), language processing (e.g., inferior prefrontal cortex), socioemotional processing (e.g., cingulate cortex, medial prefrontal cortex, hippocampus, medial temporal), multimodal integration (e.g. parietal), and planning and executing motion (e.g., premotor cortex) (Nolte, 2008).

Active music engagement enhances cognitive abilities such as memory, verbal skills, and spatial-temporal skills (Stekić, 2024). The benefits of active music engagement are further supported by studies demonstrating that lifelong musicians perform better on a wide range of cognitive domains (Sutcliffe et al., 2020) and showed less age-related volume reductions in prefrontal and inferior frontal areas than nonmusicians (Sluming et al., 2002). These findings suggest that musical training facilitates brain health. However, whether musical training in nonmusicians can preserve brain health in older age remains unclear.

The brain can be modeled as a network of regions that are spatially dispersed and functionally connected to each other (Pessoa, 2014; van den Heuvel and Pol, 2010). While brain regions may specialize in certain roles, they are continuously communicating with regions that are functionally- or structurally-related (Pessoa, 2014; van den Heuvel and Pol, 2010). Complex human behavior and cognition is increasingly thought to rely heavily on dynamic communication between regions rather than localized activity in one region (Bressler and Menon, 2010; Cohen and D’Esposito, 2016).

Whole-brain network activity can be assessed while performing a task or during resting state. In either case, functional neuroimaging data is utilized to determine the connectivity between brain regions based on similarities in their activity patterns, and the structure of the network can then be quantified and studied by a variety of indices (Lynn and Bassett, 2019; van den Heuvel and Pol, 2010). In particular, modularity and flexibility are indices rooted in complex systems theory that relate the organization of a biological system to how it functions (Simon, 1962). In the context of brain networks, modularity and flexibility are based on the functional connections between brain regions and the partitioning of brain regions into communities, known as modules. Modularity quantifies how isolated these modules are from the rest of the network on average over the duration of a neuroimaging scan (Sporns and Betzel, 2016). Higher modularity indicates that each brain region is more active with the other regions inside its module than with those outside its module, leading to more segregated cognitive processing. Lower modularity means that there are less distinct module boundaries. Flexibility quantifies the stability of these modules by looking at how module membership changes throughout the scan (Bassett et al., 2011). Higher flexibility indicates that brain regions are often changing which module they are a part of, leading to more diffuse and complex cognitive processing. Lower flexibility means that brain regions remain relatively fixed in a particular division of modules.

Given that music engagement involves a wide range of mental processes, a whole-brain network analysis is well-suited for studying cognitive health in conjunction with a music creativity intervention. Previous work with task-based functional neuroimaging during music listening has shown that there are distinct differences in neural activity, as quantified by modularity and flexibility, for familiar versus unfamiliar music (Bonomo et al., 2022). Resting-state functional imaging, in which a participant’s brain is scanned while they are not presented with any external stimulus, captures spontaneous neural activity. The functional network determined by these spontaneous activity patterns represents connections between brain regions that often work together during a variety of tasks (van den Heuvel and Pol, 2010). This would suggest that the resting-state network is more generalizable to overall brain function and health than a network captured during task performance. To the best of our knowledge, a whole-brain network analysis from resting-state data in conjunction with music engagement has not yet been conducted.

In this pilot study, we examined whether music creativity training in older adults alters functional brain networks, specifically network modularity and flexibility. We hypothesized that those in the music intervention would exhibit an increase in neural flexibility and decrease in neural modularity relative to those in the control group. In addition, because varying degrees of neural flexibility and modularity are important for engaging in complex tasks (Ramos-Nuñez et al., 2017; Yue et al., 2017), we also examined whether music intervention benefits on cognition depended on network indices at baseline. This exploratory aim would help clarify which people would benefit cognitively from music creativity interventions. Based on existing literature, we hypothesized that music intervention effects on global cognitive function would be strongest for those with higher flexibility and lower modularity at baseline.

## 2 Materials and methods

### 2.1 Study sample and design

Older adults with mild cognitive impairment or aged 70+ years old were recruited from the local community to participate in a semi-randomized controlled study examining the effects of a group music creativity intervention on cognitive function. The study is registered on ClinicalTrials.gov (NCT04137913). All participants provided informed consent and study procedures were approved by the Rice University Institutional Review Board.

#### 2.1.1 Recruitment and screening

Participants were recruited from the local community through flyers, community events, and doctor referrals. Interested participants underwent telephone screening. Inclusion criteria were as follows: Participants were (1) 70+ years old OR diagnosed with early to moderate MCI (confirmed through physician’s release of medical records using a medical release form), (2) able to read and write in English, (3) cognitively competent to participate (i.e., comprehension was assessed via three questions during the consent process), (4) demonstrate an ability to follow instructions. Exclusion criteria consisted of the following: Participants (1) had Class III heart failure, any autoimmune and/or inflammatory disorders, or Parkinson’s disease; (2) had any contraindications for undergoing functional magnetic resonance imaging (MRI) scanning (e.g., any implanted medical device, severe claustrophobia, history of working with metal, bullet wounds as determined by a standard clinical questionnaire) or had dental implants/extensive dental work that would significantly distort functional imaging data; (3) were pregnant or nursing women; (4) weighed 300+ lbs or had a body mass index that exceeded 40 (for inflammatory reasons); or (5) were a current or past professional musician. Recruitment and study enrollment took place between September 2019 and March 2023.

#### 2.1.2 Study design

Eligible participants completed two in-person assessments at baseline and at follow-up. At each in-person visit, participants completed neuropsychological testing, physical health assessments (e.g., anthropometric measurements, blood draw), self-report questionnaires, and a functional magnetic resonance imaging scan. With the exception of participants recruited between December 2020 and August 2021, participants were randomly assigned to either the music creativity group or the inactive control group and informed of their assignment at the end of the baseline visit. Participants assigned to the music creativity group completed the 6-week music creativity class on Rice University campus for 2 h a day, 3 days a week. Participants assigned to the inactive control group received no music class and were asked to refrain from joining any music-related class while enrolled in the present study. For music participants, follow-up studies took place within 0–3 weeks after the conclusion of the music class. Follow-up visits for control participants were scheduled to parallel the time gap for participants in the music creativity group: control participants completed follow-up assessments approximately 7–12 weeks after the baseline visit. Further details about recruitment and study design—such as modifications made during the COVID-19 pandemic—can be found in a previously published, feasibility and acceptability study (Wu-Chung et al., 2023).

#### 2.1.3 Music intervention class

The 6-week music intervention incorporated listening, theory, performance, and creation into each workshop in a group setting. Each music cohort consisted of 6–12 participants, and all cohorts were taught by the same musical instructor. As the course progressed, class topics became more advanced. For example, participants were introduced to familiar music during the first few weeks and were eventually exposed to symphonic movements and unfamiliar music during the last few weeks. Participants used household items, percussion instruments, and their voices to produce novel pieces. Creative compositions included but were not limited to a soundscape of a personally significant location or event, variations of a percussive rhythm, a narrative accompanied by music, and improvisation to a silent film. The course culminated with a concert on the last day, in which participants’ family and friends were invited to attend.

#### 2.1.4 Analytic sample

**FIGURE 1:**
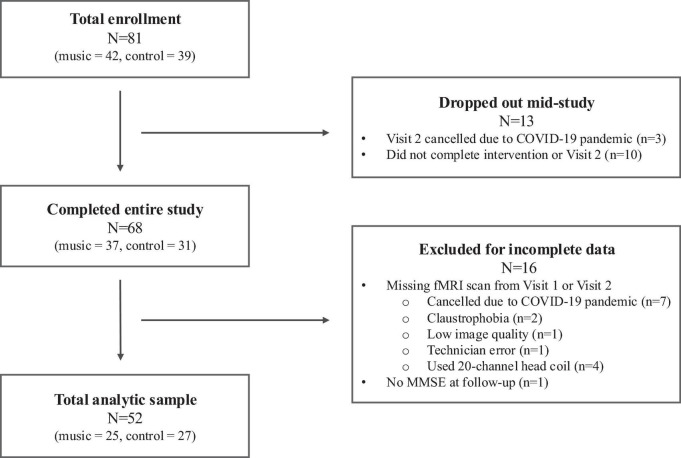
Flow chart of final analytic sample.

Only participants who completed the entire study and had valid neuroimaging scans and MMSE data from both assessment visits were included in the present analyses. Out of the 81 participants enrolled in the study, 13 dropped out before the completion of the study. Of the 68 participants who completed both visits, 11 participants did not have good quality brain scans from both visits due to a variety of reasons (e.g., public health mandates due to COVID-19 pandemic *n* = 7, claustrophobia *n* = 2, low imaging quality *n* = 1, administrator/technician error *n* = 1). We additionally excluded participants who used the 20-channel head coil instead of the 64-channel head coil (*n* = 4). One participant did not have follow-up MMSE data due to administrator error. After excluding participants for the abovementioned reasons, the final sample size for analysis was 52 (25 music, 27 control). A flow chart depicting total enrollment, exclusions, and final analytic sample is depicted in Figure 1.

### 2.2 Measures

#### 2.2.1 Brain network indices: flexibility and modularity

Functional magnetic resonance imaging (fMRI) data was acquired on a MAGNETOM 3T Vida scanner (Siemens Healthcare Limited, Erlangen, Germany) at Houston Methodist Research Institute using a 64-channel head coil. Resting-state functional scans were acquired using a T2*-sensitive echo-planar imaging (EPI) imaging sequence with the following protocol: 60 axial slices, 3 mm isometric voxels, repetition time (TR) = 2,400 ms, echo time (TE) = 35 mm, Flip angle = 70°; 216 whole-brain volumes per run, no interslice gap. A high-resolution T1-weighted anatomical scan was acquired.

Preprocessing was performed on SPM12 software (Functional Imaging Laboratory, University College London, London, England) using standard parameters: slice-timing correction, realignment, coregistration between each participant’s functional and anatomical data, normalization to a standard template, with 3-mm isometric voxels, and spatial smoothing using a 6 mm Gaussian kernel.

Following preprocessing, the functional and anatomical scans were aligned and transformed into Talairach coordinates using AFNI software (Cox, 2012). The brain was then divided into 84 Brodmann areas, and the functional signal from all voxels in each Brodmann area volume was averaged. It is worth noting that there are a variety of methods for parcellating the brain into functional and structural regions, and results from whole-brain network analyses have been shown to be consistent regardless of the chosen parcellation (Yue et al., 2017).

We considered each Brodmann area to be a network node, and the undirected links between nodes were based on their functional connections. To determine these functional connections, Pearson’s correlation coefficient was calculated on the signals for all pairs of Brodmann areas. We then filtered to keep the strongest 400 links, leading to a 11.5% network density. This filtering step reduced the presence of spurious links (Yue et al., 2017) and allowed us to make a fair comparison of brain networks across participants, since network measures are dependent on the density.

**FIGURE 2:**
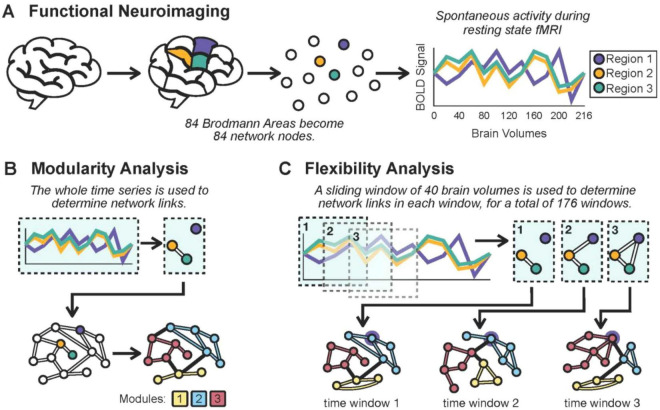
Calculation of whole-brain network flexibility and modularity from resting-state functional data. **(A)** The whole-brain is divided into 84 network nodes based on the parcellation of anatomical regions. Spontaneous activity from each brain region is captured during resting state functional neuroimaging over a run of 216 brain volumes. **(B)** For the modularity analysis, the entire time series is used to construct a whole-brain network. The correlation coefficient is used to determine network connections. In the example, Region 2 (orange) and Region 3 (green) have very similar signals throughout the entire imaging run, and so a link is drawn between them. Modularity and module membership are then determined. Modularity is defined as the percentage of network links that are between brain regions within the same module, and module membership is determined based on the groupings that would maximize modularity. In the cartoon example, there are 16 total links and 13 of which are intra-module links (color-coded according to their module), meaning 0.8 is the network modularity. The true calculation controls for the modularity value that would result from a random network. **(C)** For the flexibility analysis, a sliding window of 40 volumes is used to construct the changing whole-brain network over time. Modularity and module membership are determined for the brain network of each window. Flexibility is defined as the average rate that brain regions change their module memberships from one window to the next. In the cartoon example, brain region 1 (outlined in a purple halo) remains in the blue module for the first two time windows, but switches into the red module in the subsequent time window; among these three time windows, that brain region has a 0.5 flexibility rate.

Figure 2 depicts how we quantified modularity and flexibility from resting-state imaging data. Modularity measures the extent to which the brain is organized into groups of tightly interacting regions (Sporns and Betzel, 2016). To calculate modularity, we constructed a brain network as described above for each participant based on the signals from the Brodmann areas over the entire duration of the fMRI scan (216 brain volumes) and utilized Newman’s algorithm (Bonomo et al., 2022; Chen and Deem, 2015; Newman, 2006). The algorithm takes a top-down approach of dividing the brain regions into progressively smaller modules, and testing different module memberships, such that the final configuration maximizes the value of modularity. Modularity is calculated as the ratio of the number of within-module links divided by the total links in the network, while also controlling for the modularity that would be expected in a random network that has the same density. Importantly, the number and composition of modules are not pre-set. This data-driven approach means that module membership is not biased by any assumed functional relationships between brain regions. That being said, the resulting modules do approximately match up to expected groupings of Brodmann areas, such as brain regions involved in auditory processing, visual processing, or sensorimotor function. This is consistent with prior resting-state studies (van den Heuvel and Pol, 2010).

Flexibility measures the rate that these modules of brain regions re-organize over time (Bassett et al., 2011). To calculate flexibility, we utilized a sliding window approach (Bonomo et al., 2022; Hutchison et al., 2013). A network “snapshot” was created based on the signals from the Brodmann areas over a short window of time (40 brain volumes). This step was repeated by sliding the window one volume at a time, for a total of 176 network snapshots. The modularity algorithm described above was utilized to determine how brain regions were best partitioned into modules in each network. The modules from one window to the next were then compared, and any difference in membership of any of the brain regions was recorded. Given Newman’s algorithm may artificially designate the same module across consecutive time windows with two different labels, we used a module relabeling process devised by Ramos-Nuñez et al. (2017). A participant’s overall flexibility value was determined by averaging the number of times brain regions changed which module they were a part of, and it was scaled by the total number of regions and time windows to be a value between 0 and 1.

#### 2.2.2 Neuropsychological assessment

Global cognitive functioning was assessed using the *Mini-Mental State Exam (MMSE)*, a widely used instrument in clinical and research settings. Participants were presented with a series of questions to test orientation, registration, attention, calculation, recall, and language (Folstein et al., 1975). These included asking the participant about the current date and location, repeating three unrelated objects (and recalling them later), counting or spelling backwards, naming objects, reading and following commands, writing a spontaneous sentence, and copying geometric shapes. The number of correct answers was summed for a maximum score of 30. An extra point was given to participants who did not have a high school diploma. In this study, MMSE total scores were modeled as a continuous variable. Higher MMSE total scores indicated better global cognitive function.

#### 2.2.3 Descriptive variables

Participants self-reported their age, sex (female/male), race, ethnicity (Hispanic/Non-Hispanic), and education level. Education was categorized into 6 categories with higher values indicative of more advanced education: 0 = less than 7 years of school, 1 = 7–12 years (nongraduate), 2 = high school graduate, 3 = less than 3 years of college, 4 = 3 or more years of college 5 = graduate/professional training. Diagnosis of mild cognitive impairment was confirmed through medical records provided from the subject’s primary care physician.

### 2.3 Statistical analysis

Prior work has shown that when random assignment is used or when group assignment is not related to pre-test ability, ANCOVA, difference scores, or residual change scores are viable methods to examine pre-post change across groups (Jennings and Cribbie, 2016). Moreover, because baseline and follow-up scores are typically highly correlated, adjusting for baseline performance is recommended (Clifton and Clifton, 2019). We followed these recommendations and tested hypotheses within a residualized change linear regression framework: Time 2 dependent variable was regressed on Time 1 dependent variable to reflect residualized change from Time 1 to Time 2. We examined assumptions of linearity, normality, homoscedasticity, and multicollinearity (VIF) using diagnostic plots in R; all assumptions were met.

For primary aims, network index (i.e., modularity or flexibility) at follow-up was regressed on group assignment and baseline network index. For secondary aims, global cognitive function at follow-up was regressed on group assignment, baseline network index, baseline global cognitive function, and baseline network index × group. Simple effects using pairwise comparisons of estimated marginal means (EMM) at high (+1 SD), average, and low levels (-1 SD) of the moderator (baseline network index) were conducted to probe significant interaction effects. All analyses were run in RStudio using the following packages: ggplot2 (Wickham, 2016), arsenal (Heinzen et al., 2021), sjPlot (Lüdecke, 2021), emmeans (Length, 2022), stats (R Core Team, 2022), and apaTables (Stanley, 2020).

## 3 Results

Sample characteristics by group are depicted in Table 1. Participants in the music group had a significantly shorter follow-up period than participants in the control group (*p* < 0.01).

**TABLE 1:** Sample descriptives (*n* = 52).

	Control (*N* = 27)	Music (*N* = 25)	Total (*N* = 52)	*p*-value
Age				0.20
Mean (SD)	74.22 (4.78)	75.84 (4.15)	75.00 (4.52)	
Range	70.00–88.00	70.00–84.00	70.00–88.00	
Sex (Female)	12 (44.4%)	16 (64.0%)	28 (53.8%)	0.16
MCI diagnosis	1 (3.7%)	4 (16.0%)	5 (9.6%)	0.13
Education^a^				0.10
High school graduate	2 (7.4%)	1 (4.0%)	3 (5.8%)	
<3 years college	5 (18.5%)	1 (4.0%)	6 (11.5%)	
3+ years college	6 (22.2%)	2 (8.0%)	8 (15.4%)	
Graduate/professional training	14 (51.9%)	21 (84.0%)	35 (67.3%)	
Race^b^	2 (7.4%)	6 (25.0%)	8 (15.7%)	0.29
White	25 (92.6%)	18 (75.0%)	43 (84.3%)	
Black	1 (3.7%)	4 (16.7%)	5 (9.8%)	
Asian	1 (3.7%)	1 (4.2%)	2 (3.9%)	
American Indian/Alaskan Native	–	1 (4.2%)	1 (2.0%)	
Hispanic^b^	2 (7.4%)	3 (12.5%)	5 (9.8%)	0.54
Days between visits^c^				<0.01
Mean (SD)	80.26 (19.04)	66.32 (12.54)	73.56 (17.56)	
Range	40.00–119.00	45.00–91.00	40.00–119.00	
Baseline MMSE				0.34
Mean (SD)	27.96 (2.89)	27.20 (2.78)	27.60 (2.84)	
Range	15.000–30.00	18.00–30.00	15.00–30.00	

^a^MCI, mild cognitive impairment.

^b^N = 1 chose not to respond to race/ethnicity question.

^c^Days between baseline in-person visit and follow-up in-person visit.

### 3.1 Primary aim: examine main effect of group on change in network indices

There was no significant main effect of group on modularity (*b* = 0.01, *p* = 0.69) or flexibility (*b* = –0.01, *p* = 0.21).

### 3.2 Secondary aim: Examine baseline network index as moderator of group effect on cognitive performance

**FIGURE 3:**
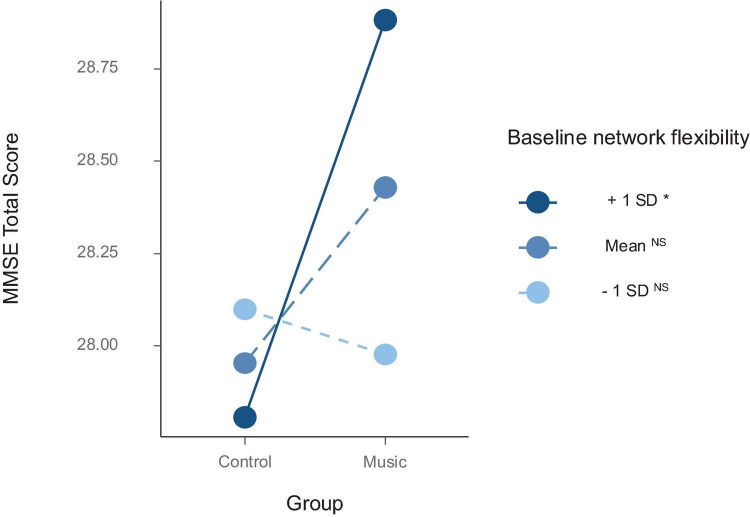
The relationship between treatment group and global cognition (MMSE) at varying levels of baseline whole-brain network flexibility. Superscript symbols in the legend indicate statistical significance of simple slopes testing at +1/Mean/-1 SD values of the moderator (i.e., baseline flexibility). *p < 0.05. NS, not significant. “MMSE Total Score” reflects regress change results (i.e., MMSE scores at follow-up after controlling for baseline MMSE).

The interaction between baseline flexibility and group significantly predicted change in MMSE scores (see Table 2; Figure 3). Simple effects tests using pairwise comparisons at high, average, and low flexibility revealed that music participants showed an increase in MMSE scores (EMM = 28.9, SE = 0.20) compared to control participants (EMM = 27.8, SE = 0.25) *only* among subjects with higher than average baseline flexibility [*t*(47) = 2.69, *p* = 0.010, Cohen’s *d* = 1.08]. No group differences in MMSE performance were observed among individuals showing average (*p* = 0.14) or lower than average baseline flexibility (*p* = 0.65).

**TABLE 2:** Residualized MMSE regressed on group, baseline flexibility, and group × baseline flexibility.

Predictors	Estimates	CI	*p*
(Intercept)	11.27	7.38–15.17	**<0.001**
MMSE (baseline)	0.64	0.54–0.74	**<0.001**
Group (Music)	–3.76	–7.99 to 0.47	0.080
Flexibility (baseline)	–5.11	–21.52 to 11.30	0.534
Group × Flexibility (baseline)	20.94	0.35–41.53	**0.046**
Observations	52		
R^2^/R^2^ adjusted	0.799/0.782		

Bolded values indicate statistical significance at *p* < 0.05.

The interaction between baseline modularity and group did not predict change in MMSE scores (*b* = 1.74, *p* = 0.74).

Models incorporating covariates can be found in Supplemental material. Importantly, when age, sex, and education were included as covariates, overall patterns remained consistent, with baseline flexibility being a significant moderator of the relationship between group and change in MMSE performance (*p* = 0.05).

## 4 Discussion

The present study examined: (1) the relationship between music creativity training and functional brain networks and (2) the relevance of brain networks for music-related improvements in global cognition. Music creativity did not alter functional neural modularity or flexibility from baseline to follow-up. However, the intervention’s benefits on global cognitive performance did depend on whole-brain network flexibility at baseline. Participants in the music creativity group showed larger improvements in global cognitive performance compared to participants in the control condition, only among those with higher-than-average baseline network flexibility. Findings suggest that neural network dynamics at baseline may be an important determinant of who may benefit cognitively from music creativity interventions.

Amidst growing curiosity in the mechanisms driving music-related cognitive plasticity (Chen et al., 2022; James et al., 2020), preliminary studies suggest that musical training modifies brain network dynamics at rest and during music listening (Faber et al., 2024; Li et al., 2019; Quinci et al., 2022). Among naïve young adults (29 music intervention participants, 27 controls), a 24-week piano training intervention, relative to no intervention, enhanced resting-state functional flexibility in visual and auditory systems and increased auditory connections within and between other functional systems (Li et al., 2019). In healthy older adults, 8-week music-listening interventions enhanced connectivity between auditory regions and the medial prefrontal cortex (*n* = 16) (Quinci et al., 2022) and increased the duration of time the temporal-mesolimbic network was engaged while listening to music (*n* = 27) (Faber et al., 2024). The relevance of neural dynamic changes to cognitive function was not reported in these studies (Li et al., 2019; Quinci et al., 2022). However, one group observed that increases in resting-state functional connectivity between the front-parietal network and default mode network following music-based neurological rehabilitation correlated with improvements in executive functioning performance in patients with traumatic brain injury (Martínez-Molina et al., 2022). Together, existing research suggests that musical training may reorganize brain networks but the extent of this change, which networks are affected, and the cognitive significance of network dynamic changes requires replication and further investigation.

Differences in study design, sample demographics, and music intervention characteristics make comparisons between the present work and existing literature difficult and likely contribute to conflicting results across studies. Indeed, while our study most closely resembles the neuroscientific approach adopted by Li et al. (2019) (i.e., resting-state functional flexibility and modularity), our participants were much older than Li’s and our music intervention focused on composing new creative pieces with percussion instruments and household objects rather than learning to read and play pre-written musical notation. Given that processing speed, attention, and executive function decline with increasing age, older adults may require longer training exposure to see long-lasting changes in whole-brain network dynamics. Indeed, prior work has found that younger patients benefited from a 10-week music intervention compared to older adults (Särkämö et al., 2016). Follow-up studies will be needed to examine whether altering the intervention design (i.e., increasing the number of courses or using alternative approaches for neuroimaging data) may produce different effects on functional networks.

In the present study, network flexibility was an important determinant of music-driven changes in cognitive function among older adults. High flexibility means the brain network has a less stable structure, with brain regions persistently changing their module allegiance. The brain network’s organization is directly tied to the brain’s efficiency and ability to integrate information (van den Heuvel and Pol, 2010). Network flexibility has previously correlated positively with cognitive flexibility, which is the ability to alter one’s behavior and thinking in changing situations (Braun et al., 2015). Whole-brain flexibility has been associated with cognitive control processes, including the ability to task switch, perform response selection, and maintain working memory (Braun et al., 2015; Ramos-Nuñez et al., 2017), and faster learning rates of visual cues (Gerraty et al., 2018). In the creativity literature, cognitive flexibility is crucial for creating new combinations from existing knowledge (e.g., autobiographical memory, episodic, and semantic memory) and emotion (Dietrich, 2004). Creating musical pieces based on new concepts learned each week was central to the music creativity curriculum implemented in the present study. Thus, individuals with flexible thinking patterns or an “open mind” may learn faster and be better able to integrate new concepts at subsequent classes than those with rigid thinking patterns. Previously, higher network flexibility correlated with the tendency to engage in effortful, cognitive endeavors, and this flexibility mediated the relationship between the tendency to engage in cognitive endeavors and creative achievement (He et al., 2019). While it would be premature to conclude that higher network flexibility reflects higher cognitive flexibility, our findings do suggest that flexible whole-brain networks are important for understanding individual differences in music-induced cognitive change.

We originally postulated that participants with both high baseline flexibility and low baseline modularity would exhibit the largest gains in global cognitive function. The fact that modularity was not predictive of the cognitive benefit from the music study may be related to modularity and flexibility reflecting different cognitive processes that were not equally targeted by the activities of our music creativity intervention. Indeed, previous work has shown that, while high flexibility correlated with better performance on complex tasks, low modularity did not significantly correlate with better performance on these same tasks (Ramos-Nuñez et al., 2017; Yue et al., 2017). Modularity and flexibility as network measures are not inherently negatively correlated with each other based on how they are calculated (Ramos-Nuñez et al., 2017). Furthermore, they have even exhibited a significant null correlation with each other during task-based fMRI while participants listened to culturally unfamiliar music (Bonomo et al., 2022). Given network indices in the present study were determined from resting-state neuroimaging, it is unclear what aspects of the intervention (i.e., music listening, theory, performance, and creation) may have specifically exploited high flexibility and not low modularity. Future work utilizing task-based neuroimaging during music creativity engagement may clarify the different cognitive processes that modularity and flexibility may support.

This study contains some limitations. Patterns were observed in a small sample which may limit the generalizability of our findings. Future studies will be important for validating these preliminary findings in a larger sample. Second, participants in this study were highly educated, with most receiving post-graduate level degrees. In addition, due to the time commitments of the study, only participants who could commit to a 6-week in-person intervention were deemed eligible; this selection process may inherently bias the sample towards those who are healthier, more financially stable, more socially integrated (and able to acquire access to transportation), and self-motivated. Future work will be necessary to ascertain whether network indices are relevant for differentiating music-related cognitive outcomes among individuals of varied socioeconomic and health backgrounds. Third, while dynamic network indices are hypothesized to reflect complex cognitive processes, the functional relevance of these network indices, such as how network indices correspond to relevant cognitive outcomes and how stable these network patterns are across contexts and individuals, is not certain and remains an ongoing topic of investigation. Lastly, patterns were observed relative to an inactive comparison group. Because some effects may be attributed to extant features of the intervention (e.g., behavioral activation, social engagement) rather than the intervention itself, future work should examine whether findings remain consistent when comparing the music intervention to an active control condition.

Several strengths should be noted. As a randomized controlled trial, this study was conducted within a controlled environment to minimize confounders and provide robust evidence of causality. To minimize group selection bias, participants were randomized into the music or control group, expressed their willingness to commit to the study regardless of group assignment, and informed of their group assignment at the conclusion of their first in-person visit. To maintain consistency across all music cohorts, all music classes were taught by the same music instructor. Both music and control groups received the same neuroimaging and neuropsychological testing protocol. Music class attendance rates were high; the average attendance rate was 86.59% and 80% of music participants attended 89% or more of the classes.

The benefits of the musical arts on cognitive aging have become increasingly evident (Fusar-Poli et al., 2018; Wu-Chung et al., 2023; Xu et al., 2017). Yet, the mechanisms underlying music-driven changes in cognition have been less well-studied. Using a randomized clinical trial design, we demonstrated that high neural network flexibility was necessary to experience the cognitive benefits of engaging in a music creativity intervention. At the same time, the 6-week music intervention did not alter whole-brain network indices from baseline to follow-up. Findings suggest that network organization, specifically the brain’s tendency to reorganize into different modules, explains individual differences in music-induced cognitive change. As one of few experimental studies investigating the neuroscience of music creativity and cognition in older adults, this pilot study highlights the importance of brain networks for preserving cognitive health in older adulthood. Future studies with larger sample sizes are needed to clarify whether these patterns are replicable and generalizable to diverse populations.

## Data Availability

The raw data supporting the conclusions of this article will be made available by the authors, without undue reservation.
