# Supplementary material for: Music-induced cognitive change and whole-brain network flexibility: a pilot study
Source: Front Neurosci. 2025 Jun 5;19:1567605. doi: 10.3389/fnins.2025.1567605 (PMC12176889; doi:10.3389/fnins.2025.1567605)
Supplement: Supplementary file 1 [file Table_1.docx]

Table S1. Residualized MMSE regressed on baseline flexibility, group assignment, and covariates

|  | **MMSE at follow-up** | | | | | |
| --- | --- | --- | --- | --- | --- | --- |
| *Predictors* | *Estimates* | *CI* | *P* | *Estimates* | *CI* | *p* |
| (Intercept) | 10.03 | 3.04 – 17.02 | **0.01** | 12.34 | 4.80 – 19.88 | **<.01** |
| MMSE (baseline) | 0.63 | 0.52 – 0.74 | **<0.001** | 0.60 | 0.47 – 0.73 | **<0.001** |
| Group [Music] | -3.69 | -7.86 – 0.48 | 0.08 | -3.30 | -7.78 – 1.17 | 0.14 |
| Flexibility (baseline) | -3.73 | -20.01 – 12.55 | 0.65 | -2.15 | -20.97 – 16.68 | 0.83 |
| Education | -0.17 | -0.47 – 0.14 | 0.28 | -0.14 | -0.45 – 0.18 | 0.39 |
| Age | 0.02 | -0.05 – 0.09 | 0.50 | -0.01 | -0.09 – 0.07 | 0.82 |
| Female | 0.66 | 0.09 – 1.23 | **0.02** | 0.59 | -0.07 – 1.24 | 0.08 |
| Group × Flexibility (baseline) | 20.23 | -0.01 – 40.48 | **0.05** | 19.89 | -2.22 – 41.99 | 0.08 |
| Days between visits |  |  |  | 0.01 | -0.01 – 0.03 | 0.49 |
| Race [Non-White] |  |  |  | -0.75 | -1.64 – 0.13 | 0.09 |
| Observations | 52 | | | 51 | | |
| R^2^ / R^2^ adjusted | 0.83 / 0.80 | | | 0.84 / 0.80 | | |

Note: ‘Education’ was modeled continuously to minimize loss of degrees of freedom. Race was modeled as a binary variable (0 = White, 1 = Non-White) due to low numbers within each non-White subgroup of Race: Asian, Black, and American Indian/Alaskan Native.
